# Niosome: A Promising Nanocarrier for Natural Drug Delivery through Blood-Brain Barrier

**DOI:** 10.1155/2018/6847971

**Published:** 2018-12-11

**Authors:** Mahmoud Gharbavi, Jafar Amani, Hamidreza Kheiri-Manjili, Hossein Danafar, Ali Sharafi

**Affiliations:** ^1^School of Pharmacy, Zanjan University of Medical Sciences, Zanjan, Iran; ^2^Applied Microbiology Research Center, Systems Biology and Poisonings Institute, Baqiyatallah University of Medical Sciences, Tehran, Iran; ^3^Zanjan Applied Pharmacology Research Center, Zanjan University of Medical Sciences, Zanjan, Iran

## Abstract

Niosomes (the nonionic surfactant vesicles), considered as novel drug delivery systems, can improve the solubility and stability of natural pharmaceutical molecules. They are established to provide targeting and controlled release of natural pharmaceutical compounds. Many factors can influence on niosome construction such as the preparation method, type and amount of surfactant, drug entrapment, temperature of lipids hydration, and the packing factor. The present review discusses about the most important features of niosomes such as their diverse structures, the different preparation approaches, characterization techniques, factors that affect their stability, their use by various routes of administration, their therapeutic applications in comparison with natural drugs, and specially the brain targeting with niosomes-ligand conjugation. It also provides recent data about the various types of ligand agents which make available active targeting drug delivery to the central neuron system. This system has an optimistic upcoming in pharmaceutical uses, mostly with the improving availability of innovative schemes to overcome blood-brain barrier and targeting the niosomes to the brain.

## 1. Introduction

Several brain and CNS diseases such as neurological diseases (meningitis, encephalitis, viral, bacterial, protozoan, and fungal and worm infections), neurological disorders (epilepsy, seizures, trauma, Parkinson, multiple sclerosis, dementia, Alzheimer, mononeuropathy, polyneuropathy, and myopathy), and brain tumors (cerebral tumors and glioma) are associated with mortality. These problems needed proper drug delivery for treatment [1]. Several approaches to create novel CNS drug-delivery systems are primarily due to the anatomical and physiological characteristics of the blood-brain barrier (BBB) [2–4]. Neural tissues of the brain are protected in contradiction of neurotoxic agents and variation in blood structure that are important for regular purpose of the neurons that covered through BBB. Most organs in our body, apart from the brain and spinal cord, are perfused by capillaries lined with endothelial cells which need small pores to let the small molecules move fast into the organ interstitial fluid from the circulation [5]. In the brain arteriole, ECs are connected to each other by continuous tight junctions (TJs), known as zonula occludens, which cover the paracellular pathway [6, 7]. This can efficiently block the free polar solutes from paracellular pathways and so cast off admission to brain interstitial fluid. Therefore, the BBB let the small particles to break over the brain through the blood stream such as lipophilic solutes or those that pass in the brain by an active transport apparatus, mainly with crucial nutrients, precursors, and cofactors [8–11]. BBB can be transported into the brain endothelium by several mechanisms, such as BBB peptide transport mechanisms. Previous studies suggest that this mechanism is the principal attitude for drug delivery to the brain. Generally, there are three systems for drug delivery to the brain [8, 12] including systemic absorption through BBB and nasal and intracerebroventricular (ICV) administration. On the other hand, each one of these methods has several disadvantages which are listed below.

Disadvantages of systemic absorption through the BBB are given below [2, 8]:

1. Systemically administered therapies may fail to reach therapeutic levels in the CNS.
2. In some cases, intravenous therapy may cause systemic toxicity.
3. In neurodegenerative disease, BBB efficiency decreases. It may cause brain vascular damage as well as initiating BBB dysfunction or reducing of blood carriage into the brain which obstacles drug delivery into the brain. This also follows through a chronic medical condition called hypoxia.

Disadvantages of nasal administration are given below [13, 14]:

1. May cause irritation to the nasal mucosa
2. Nasal congestion as the result of allergies may obstacle absorption of the drug
3. Drug delivery efficiency decreases as molecular weight increases
4. Excessive use of this method causes mucosal damage

Disadvantages of ICV administration are given below [15–17]:

1. ICV administration requires a device entrenched by neurosurgeons in the subgaleal space under the scalp and associated with the ventricles inside the brain through an outer catheter.
2. High intracranial pressure throughout drugs' administration using the ICV method; this is the case particularly after higher volumes are directed in excess of a short period. This can cause the patient endure risky and even intolerable pain.

However, as it is mentioned above, systemic absorption through the BBB is easier than the other methods. To suggest an alternative drug-delivery system, two provisions must be deliberated. The drug must be released in a steady rate; it must release in an adequate quantity of the active component at the desire site. The previous methods do not chance these requirements. To accomplish these requirements, nanostructures are a promising approach to improve natural drug delivery through the brain.

The nanostructure could change the characteristics and the behavior of the natural drugs inside the body after administration. It can protect natural drugs from degradation [18, 19] and in delivering them to their target sites [19]. Also, prolongation of blood circulation time [20], enhancement of drug accumulation in the pathological tissues [21], and decreasing toxicity can organize the application of the nanostructure for numerous pharmaceutical uses [22]. On the other hand, drug delivery efficiency can be increased through ligand bindings and applying the natural drug in different surfaces of the body. This is performed by passive diffusion which is contingent on lipophilicity and molecular weight or through active transport systems by interacting with the blood components having the role of a mediator between the blood carrier and the brain. Nanostructures behave differently depending on the surface area and the ligand bindings as well as its mediator [23, 24]. This is typical in treating pathological diseases such as glioblastoma and neurodegenerative diseases. Depending on the biomaterial and morphology of the drug-delivery system, various nanoparticles can be prepared from polymers, metals, nanogel and colloidal systems, and particular and vesicular systems. Vesicular systems include vesicular drug-delivery system that has covering liposomes, ethosomes, transfersomes, bilosomes, and niosomes [25, 26]. Amongst these systems, particularly, liposomes and niosomes are used in treating pathological disease whose sufficiency can be enhanced by targeting permeable components passing through tissues via blood vessels [27–29]. This method has high efficiency compared with the reticuloendothelial system (RES) that could be dysfunctional by removing vesicular particle from the plasma. The important part of applying a successful drug delivery to the brain is performing through increasing the circulation time. This review will focus on niosomes as nanoparticles that are designed for improving their medicinal purposes and consequently to overcome BBB and procedures to progress natural drug delivery efficiency.

## 2. Structure and Components of Niosomes

### 2.1. Components of Niosomes

The two major components utilized for the preparation of niosomes exist: lipid compounds (cholesterol or L-*α*-soya phosphatidylcholine) and nonionic surfactants. Lipid compounds are utilized to provide unbending nature, appropriate shape, and adaptation to the niosomes [30]. The part surfactants assume the main part in the development of niosomes. The accompanying nonionic surfactants for the most part utilized for the arrangement of niosomes are the spans (spans 60, 40, 20, 85, and 80), tweens (tweens 20, 40, 60, and 80), and Brij (30, 35, 52, 58, 72, and 76) [31–33]. Nonionic surfactant-based vesicles or niosomes are the capable drug carriers which require a bilayer structure that are made mostly by nonionic surfactant and lipid compounds (cholesterol or L-*α*-soya phosphatidylcholine) incorporated in an aqueous phase.

#### 2.1.1. Nonionic Surfactant

Niosomes are multilamellar vesicles prepared from synthetic nonionic surfactants. The nonionic surfactant has a hydrophilic head group and a hydrophobic tail which affect the entrapment efficiency of the drug. As the HLB value of surfactant increases, therefore, alkyl chain rises, thereby, the size of niosomes rises. Therefore, HLB rate 14–17 is not suitable for niosomes formulation [34, 35]. Beyond amount of surfactant, the surfactant structure played main role for stability and privation vesicle aggregation of niosomes by repulsion of steric or electrostatic force [35]. The effect of surfactant's structure in niosomes formation explains with critical packing parameter (CPP) that definite with the following equation [36]:

(1) $$\begin{array}{c} \text{CPP}=\frac{V}{I_{\text{c}}}\times A_{\text{o}}, \end{array}$$

CPP is the critical packing parameter, *V* is the hydrophobic group volume, *I*_c_ is the critical hydrophobic group length, and *A*_o_ is the area of the hydrophilic head group. The type of micellar structure was predicted by the critical packing parameter value as assumed:

  If CPP <1/2 formation of spherical micelles
  If 1/2 < CPP <1 formation of bilayer micelles
  If CPP >1 formation of inverted micelles

Several sorts of surfactant are applied in preparation for niosomes such as alkyl ethers and alkyl glyceryl ethers, sorbitan fatty acid esters, polyoxyethylene fatty acid esters, and block copolymer (pluronic L64 and pluronic p105). To achieve these structures, some input energy, for example, mechanical (stirring or sonicates) or heat is required.

#### 2.1.2. Alkyl Ethers and Alkyl Glyceryl Ethers

Alkyl ethers are good vesicle-forming nonionic surfactants. They are stable, relatively nonallergic to skin and compatible with other surfactants [37]. Because of their great constancy, they can be applied to encapsulate peptides and proteins [38].

**Figure 1:**
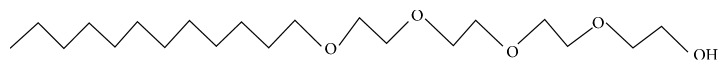
Brij 30.

*(1) Polyoxyethylene 4 Lauryl Ether (Brij 30)*. Brij 30 has an HLB value of 9.7 and a phase transition temperature of <10°C [39, 40]. Unlike other alkyl ether derivatives, that reduce vesicle formation in the presence of cholesterol, Brij 30 formed large unilamellar vesicles when combined with 30 mmol/L cholesterol. Nevertheless, it is discordant with benzocaine, tretinoin, and oxidizable medications; meanwhile, with such substances, it causes oxidation leading to discoloration of product. This surfactant does not suit properties to apply for formulation of some drugs and iodides, mercury salts, phenolic ingredients, salicylates, sulfonamides, and tannins (Figure 1).

*(2) Polyoxyethylene Cetyl Ether (Brij 58)*. Brij 52, 56, and 58 are cetyl derivatives of polyoxyethylene that can be used for vesicle formation.

**Figure 2:**
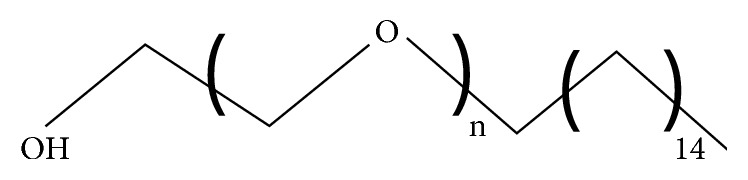
Brij 58.

Among them, Brij 58 has developed because of its capacity to arrange inverse vesicles, which are suitable for possible pharmacological requests. The HLB value of Brij 58 remains 15.7 [39] (Figure 2).

*(3) Polyoxyethylene Stearyl Ethers (Brij 72 and Brij 76)*. These are some derivatives of polyoxyethylene ether with worthy vesicle-forming possessions. Especially, Brij 72 and Brij 92 can be used to form multilamellar vesicles with high encapsulation effectiveness which are higher than Brij 76 because of low HLB = 4.9 compared to Brij 76 = 12.4 [39, 41].

#### 2.1.3. Sorbitan Fatty Acid Esters

These are some products of polyoxyethylene esters that are mostly applied in maquillages in water-based products. Sorbitan esters are frequently mentioned to as spans. Their gel transition temperature rises as the length of the acyl chain increases. Hence, sorbitan monolaurate (Span 20) with a C9 chain has a liquid transition at 24°C; sorbitan monopalmitate (Span 40) with a C13 chain has a gel transition temperature of 46-47°C; sorbitan monostearate (Span 60) with a C15 chain has a gel transition temperature of 56–58°C. Vesicles made with these higher molecular weight spans are principal to fewer permeable and more stable to osmotic grades [42]. The molar ratio of cholesterol to span and length of the lipophilic were critical factors for entrapment of drugs into niosomes [35]. Thus, greater encapsulation of acyclovir was described in niosomes that was made using a cholesterol (span 80 ratio of 1 : 3) [43] although high encapsulation of colchicine and 5-fluorouracil was stated in niosomes prepared by cholesterol (span ratio of 1 : 1) [44]. Fang et al. [45] reported that Span 40 was essential in a proniosomal formulation of estradiol to improve its infusion through the skin. A decline in setup efficiency of retinyl palmitate was described as the length of the lipophilic chain increase in the order of Span 40, Span 60, and Span 85.

#### 2.1.4. Polyoxyethylene Fatty Acid Esters

Polysorbates are oily liquids derived from ethoxylated sorbitan esterified with fatty acids. Mutual trade names for polysorbates contain Scattics, Alkest, Canarcel, and Tween. Tweens 20, 40, 60, and 80 are mutual polysorbates which are applied for niosomes' construction [31, 32].

#### 2.1.5. Pluronic L64 and Pluronic p105

Pluronic is a water-soluble nonionic surface-active agent, in which the triblock construction contains polyethylene oxide (PEO) and polypropylene oxide (PPO) segments with the PPO block in the middle and PEO block of equal lengths on either side of the PPO block [46]. Pluronic is arranged in a linear EO-PO-EO triblock copolymer structure. The pluronic L64 surfactant through a structural formulation of EO_13_PO_30_EO_13_ and the molecular weight of 2900 g·mole^−1^ also and pluronic P105 surfactant by a structural formulation of EO_37_PO_56_EO_37_ and the molecular weight of 6500 g·mole^−1^ were incorporated to form niosomes [47, 48].

#### 2.1.6. Cholesterol

**Figure 3:**
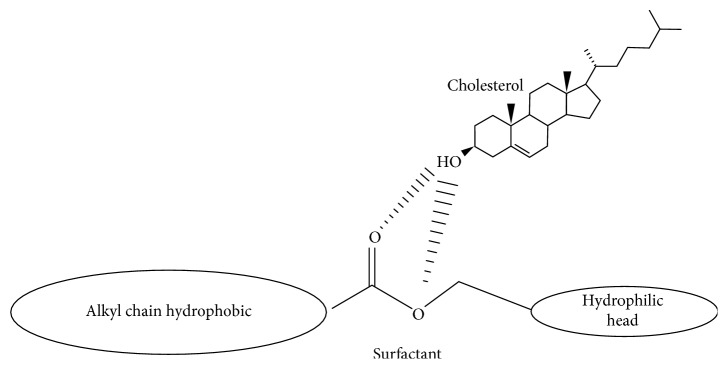
Schematic structural interaction between surfactant and cholesterol.

In the niosomes structures, cholesterol is an amphiphilic compound that can cooperate with surfactant to construct hydrogen bonding among hydroxyl groups of cholesterol with hydrophilic head of the surfactant. This results in improvement in the mechanical rigidity of vesicles and membrane cohesion and the leakiness of membrane and finally increases the entrapment efficiency of the niosomes. Cholesterol amount in niosomes influences the structures of niosomes and physical possessions and affects the entrapment efficiency, time circulation, and release of payload. According to the previous studies, it is revealed that the use of cholesterol in preparation of niosomes and its quantities is required to be adjusted depending on the physical and chemical features of surfactants and the future medicines' type. The interface of cholesterol with surfactant in the bilayer of niosomes is because of hydrogen bonding (Figure 3**)**.

#### 2.1.7. Charge-Inducing Molecule

Some charged molecules are added to niosomes for increasing the steadiness of niosomes through electrostatic repulsion which avoids aggregation and coalescence. The negatively charged molecules applied in niosomes arrangements are diacetyl phosphate (DCP) and phosphatidic acid. Stearylamine (STR) and stearyl pyridinium chloride are the famous positively charged molecules applied in niosomes construction. 2.5–5 molar % concentration of charged molecules is acceptable as high concentration can prevent the niosomes creation.

#### 2.1.8. Hydration Medium

Phosphate buffer at different pH values is frequently used in the hydration medium for the construction of niosomes. The selected pH of the hydration medium is contingent on the solubility of the medicine being encapsulated. Thus, pH 5 phosphate buffer is considered in the preparation of ascorbic acid niosomes [49], whereas pH 7 phosphate buffer is applied in the preparation of aceclofenac niosomes [48].

#### 2.1.9. Structure of Niosomes

**Figure 4:**
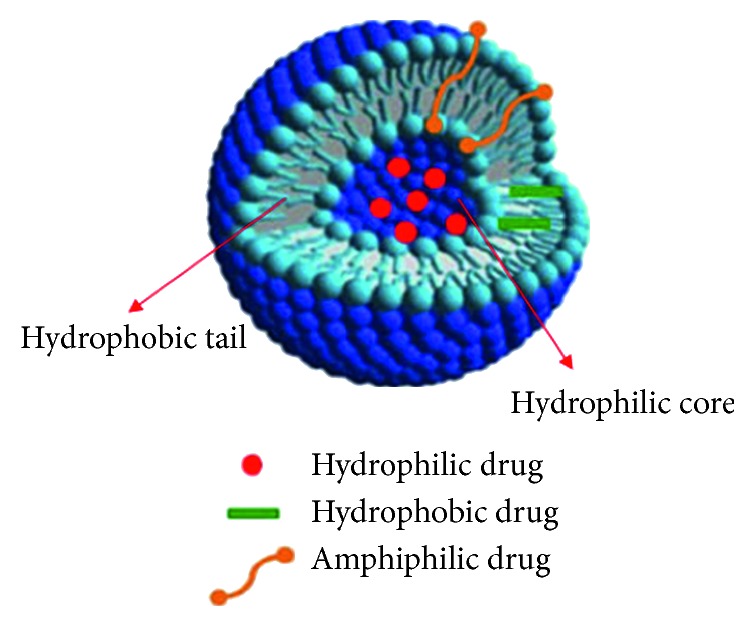
Schematic representation of a niosomes as drug-delivery system [50].

Niosome structures are made on the admixture of surfactant and cholesterol with following hydration in water. The bilayer in niosomes is prepared for a nonionic surfactant with its hydrophilic ends exposed on the outside and inside of the vesicle, while the hydrophobic chains express each other within the bilayer. As shown in Figure 4, because of high interfacial tension between water and the hydrophobic tail, monomer units aggregate into vesicle, which forms as closed bilayer structures. In order to achieve these structures, some contribution energy, for example, mechanical (stirring or sonicates) or heat, are essential. Therefore, the vesicle holds hydrophilic drugs within the space surrounded in the vesicle and hydrophobic drugs are entrapped within the bilayer itself, while amphiphilic drugs are consistent with drugs lipophilicity fixed in the space between hydrophilic core and lipophilic tail (Figure 4).

### 2.2. Comparison of Liposomes and Niosomes

**Figure 5:**
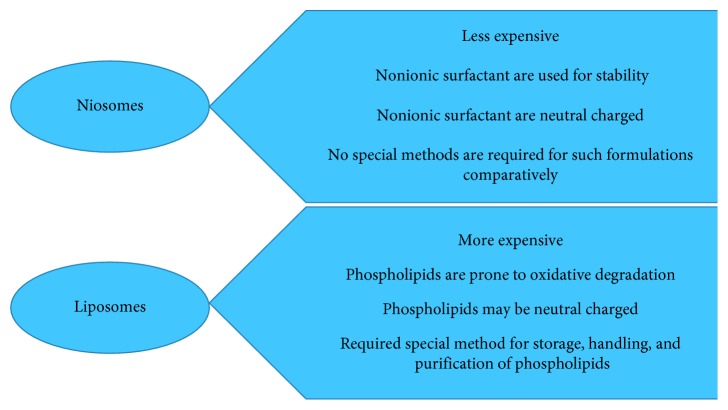
Major differences in characteristics between liposomes and niosomes.

Although the fact that the liposomes and niosomes are practically same, both can be employed as part of the focused and managed sedate conveyance framework; property of both relies on structure of the bilayer and strategies for their planning and both enhanced bioavailability and prevention the body leeway. Niosomes are organized from uncharged single-chain surfactant and cholesterol, while liposomes are organized from double-chain phospholipids, and there are major differences in features which exist between liposomes and niosomes (Figure 5).

### 2.3. Types of Niosomes

**Figure 6:**
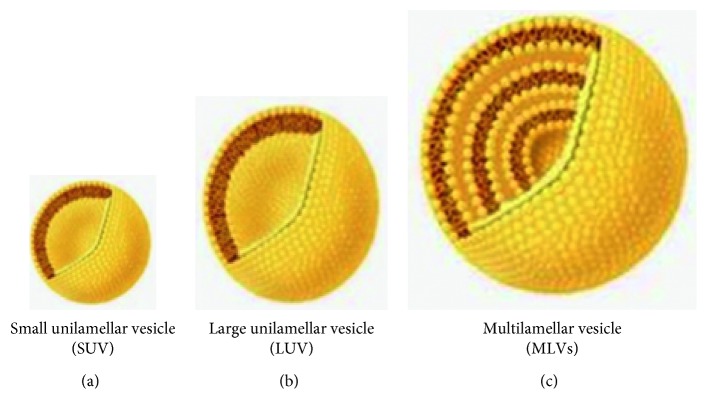
Schematic typical vesicle size of niosomes.

Types of niosomes are classified according to three factors: first, basis of function of niosomes size, second, the method of preparation, and third, based on the vesicle size. So, niosomes can be separated to three clusters including small unilamellar vesicles (SUVs, size = 0.025–0.05 *μ*m), multilamellar vesicles (MLVs, size ≥0.05 *μ*m), and large unilamellar vesicles (LUVs, size ≥0.10 *μ*m), which are described in the following subsections (Figure 6).

#### 2.3.1. Multilamellar Vesicles (MLVs)

As shown in Figure 6, MLVs are formed from some bilayers adjacent to the aqueous lipid section individually. The estimated dimensions of these vesicles stay between 100 and 1000 nm in diameter. Multilamellar vesicles, because of simple preparation, are reflexively stable upon keeping for extend phases, and appropriate for lipophilic agents, are widely used.

#### 2.3.2. Large Unilamellar Vesicles (LUVs)

The approximate sizes of these vesicles are 100–250 nm in diameter. LUV has a high aqueous part to lipid section proportion, so that the bioactive resources can be captured by membrane lipids.

#### 2.3.3. Small Unilamellar Vesicles (SUVs)

The approximate sizes of small unilamellar vesicles are 10–100 nm. Small unilamellar vesicles are consisted of several procedures, such as sonication, high-pressure homogenization, and extrusion methods.

#### 2.3.4. Bola-Surfactant Containing Niosomes

In these kinds of niosomes, bola-surfactant compounds require two hydrophilic heads which can link by one or two long lipophilic spacers. The surfactant use in bola-surfactant containing niosomes is prepared of omega hexadecyl-bis-(1-aza-18 crown-6) (bola surfactant): span-80/cholesterol in 2 : 3 : 1 molar percentage.

#### 2.3.5. Proniosomes

**Figure 7:**
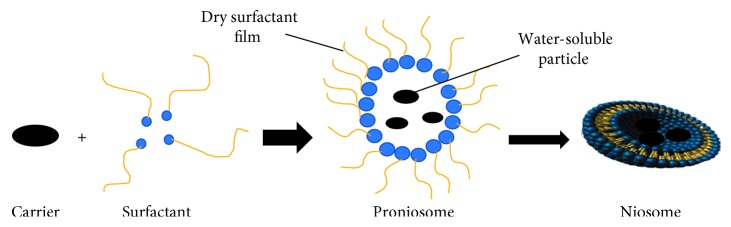
Schematic proniosome and niosomes formation process.

As shown in Figure 7, proniosomes are the niosomes formation that consists of water-soluble carriers and surfactants. The proniosomes are dehydrated niosomes constructions which would be hydrated for earlier usage. Proniosomes can decrease niosomes problems, for example, aggregation, fusion, and leakage of medication in after a while.

#### 2.3.6. Apsasome

Apsasome includes cholesterol, ascorbyl palmitate, and highly charged lipid such as dihexadecyl phosphate (DCP). It is hydrated by water solvent and sonicated to produce the final product. Apsasome can improve the transdermal drug-delivery systems and decrease the disorders which triggered using reactive oxygen species.

#### 2.3.7. Discome

Large disk-shaped structures or discomes have low cholesterol concentration. It was reported that niosomes were prepared from incubating in cholesteryl poly-24-oxyethylene ether (Solulan C24) at 75°C for 1 h to obtain spherical niosomes. This has caused in the construction of large size approximately 11–60 *µ*m and multilayered vesicular structures. Discomes act as potential drug delivery carriers as sustained release system at the ocular site.

#### 2.3.8. Elastic Niosomes

This type of niosomes could be supple lacking destroying construction, so they have the ability to permit from side to side pores in smaller their size. These vesicles have nonionic surfactants, water, and ethanol. This flexible structure can be used to increase penetration intact skin layers.

#### 2.3.9. Polyhedral Niosomes

This type of niosomes are created by hexadecyl diglycerol ether (C_16_G_2_), replacing with any of the nonionic surfactants and polyoxyethylene 24 cholesteryl ether (C_24_), without cholesterol. These vesicles have unconventional structures which can entrap water-soluble particles. Accumulation of an equimolar volume of cholesterol to the definite surfactant upsurges the curving of the membranes. These conditions result in the formation of spherical vesicles and tubules.

#### 2.3.10. Vesicles in Water and Oil System (V/W/O)

Vesicles in water and oil system contain niosomes in water in oil (as external phase) emulsion (v/w/o). This phenomenon is formed by the suspension of niosomes figured from blend of sorbitol monostearate, cholesterol, and solulan C24 (poly-24-oxyethylene cholesteryl ether) to oil phase at 60°C. This results in the formation of vesicle in water in oil (v/w/o) emulsion using cooling to room temperature forming vesicle in water in oil gel (v/w/o gel). This type of niosomes were hired for protein drug delivery and protection from enzymatic degradation after oral administration and controlled release.

#### 2.3.11. Niosomes in Carbopol Gel

In this system, niosomes were prepared from the drug, nonionic surfactant, and cholesterol; then, it is combined in carbopol-934 gel (%1 w/w) base comprising propylene glycol (%10 w/w) and glycerol (%30 w/w).

### 2.4. Advantages of Niosomes

The application of lipid vesicles and nonionic surfactant vesicles systems for therapeutic goal may suggest advantages as follows:

- Niosomes are patient compliance, biodegradable, biocompatible, nonimmunogenic, and low toxicity
- They are osmotically active and have long storing period
- They perform as a pool to release medication in a steady, organized, and sustained mode
- They provide accommodations for drug molecules with a varied sort of solubility of medication, for example, hydrophilic and lipophilic in addition to amphiphilic medication moieties
- Niosomes can rise the stability of the encapsulated medication
- Niosomes can improve the skin penetration of medications
- Niosomes have the capability to overcome BBB and access drug delivery to the brain
- They improve the therapeutic performance of the drug by surface modification and restricting effects to target cells, thereby reducing the clearance of the medication
- Niosomes can expand the oral bioavailability of medications
- Surface modification is very simple due to functional groups on their hydrophilic heads
- The characteristics of the vesicle formulation, for example, size, lamellarity, surface charge, concentration, and drug sting, are controllable
- Handling, storage of surfactants, and preparation of noisome do not require special conditions
- Simple methods are needed for manufacturing and large-scale production of niosomes

### 2.5. Limitation of Niosomes Drug-Delivery System

Although the used surfactants require further compatibility and low toxicity than other sorts of surfactants, there are not enough studies on the toxicity of niosomes. Previous studies have shown that rise in alkyl chain length of them can result in a reducing in toxicity, while rise in the polyoxyethylene chain length increases the toxicity. The highest restrictions of niosomes in drug delivery are concluded as follows:

- The aqueous suspension of niosomes could require inadequate shelf life due to combination, aggregation, permeability of captured medications, and hydrolysis of encapsulated medications
- The preparations of multilamellar vesicles are time-consuming and need distinct tools

### 2.6. Preparation Methods of Niosomes

The general method of niosomes preparation is by hydration of nonionic surfactants using hydration medium. However, they are prepared by several techniques, such as, transmembrane pH gradient method, lipid layer hydration, reversed-phase evaporation, EER injection, bubbling of nitrogen, sonication, the enzymatic method, the single-pass technique, and microfluidization which are defined here in depth.

#### 2.6.1. Transmembrane pH Gradient Method

**Figure 8:**
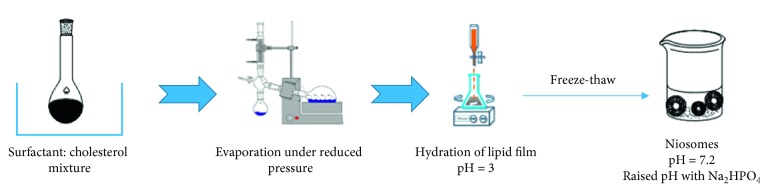
Schematic nonionic surfactant vesicles (niosomes) formation by transdermal pH gradient method.

Surfactant and cholesterol are ready in chloroform and evaporated under reduced pressure and stream of *N*_2_ to yield a tinny lipid film on the wall of a round-bottomed bottle. The obtained lipid film is hydrated with an acidic compound (usually citric acid). The resulting preparation (multilamellar vesicles) is exposed to freeze-thaw cycles [51–53]. The pH of the sample is then elevated to 7.2 (Figure 8). Bhaskaran and Lakshmi [54] reported that niosomes can be made by this process (entrapment efficiency (EE) = 87.5%).

#### 2.6.2. Lipid Layer Hydration

**Figure 9:**
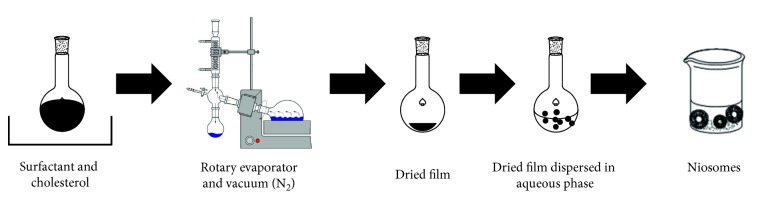
Schematic nonionic surfactant vesicles (niosomes) formation by lipid layer hydration method.

As shown in Figure 9, surfactant and cholesterol are dissolved in chloroform and evaporated under reduced pressure to produce a thin lipid film on the wall of a round-bottomed flask. The obtained film was hydrated with an aqueous solution of drug at a temperature slightly above the phase transition temperature of the surfactants under moderate shaking conditions [54–57]. Several variables were validated that comprise the mass per batch, angle of evaporation, rotation speed of the vacuum rotary evaporator, and the hydration procedure. The latter variable was developed by various solvents (water, phosphate buffer (PB), and PB/drug) and hydration temperature below and above the gel transition temperature. Sathali and Rajalakshmi prepared terbinafine niosomes by thin film hydration and settled this procedure which, upon sonication, produced small unilamellar niosomes (EE = 85%) [57].

#### 2.6.3. Reversed-Phase Evaporation

The surfactants are dissolved in a mixture of ether and chloroform and added into water phase having the medication emulsified to get w/o emulsion. The resulting mixture is homogenized, and then, organic phase is evaporated [54]. The lipid or surfactant forms a gel first and then hydrates to form spherical stable uniform vesicles [58, 59].

#### 2.6.4. Ether Injection

The mix of surfactant, cholesterol and drug, is dissolved in diethyl ether and over a gauze needle injected gradually into an aqueous phase. The ether solution is evaporated by rotary evaporator above the boiling point of the organic solvent. The large unilamellar vesicles, after evaporation of the organic solvent, are additionally exposed to decrease the size to give single-layered vesicles [58].

#### 2.6.5. Bubbling of Nitrogen

**Figure 10:**
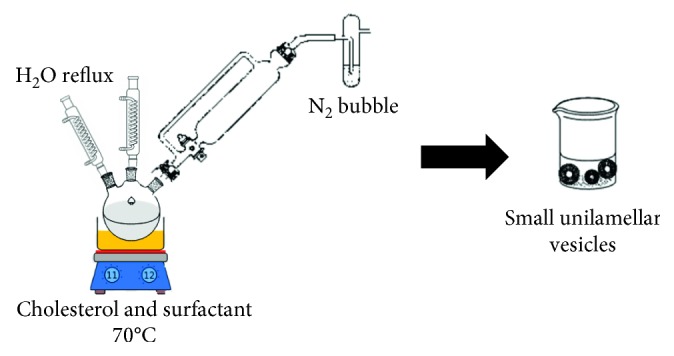
Schematic small unilamellar vesicles (niosomes) formation by bubbling of nitrogen method.

This method is a new procedure for the one-step establishment of niosomes lacking the usage of any organic solvents. Using this buffer, cholesterol and surfactant are spread together (pH 7.4) at 70°C conditions. It presumed by round-bottomed flask with three necks. The first two necks are placed in water-cooled reflux to control the temperature. Due to the sample (cholesterol and surfactant) of homogenized, nitrogen gas was passed from the third neck. Thereby, large unilamellar vesicles were produced. A continuous stream of nitrogen gas bubbles is made and introduced through the dispersion and to give small unilamellar vesicles (Figure 10) [60].

#### 2.6.6. Sonication

**Figure 11:**
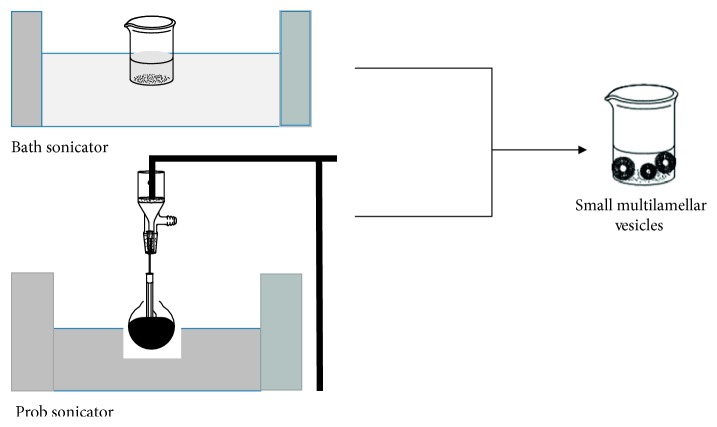
Schematic small unilamellar vesicles (niosomes) formation by sonication method.

In the sonication-mediated procedure, niosomes were prepared by Baillie et al.'s method [61]. The surfactant cholesterol combination is distributed in water phase that contains the drug in flax. The mixture is subjected to probe sonication or bath sonicator for 3 minutes at 60°C until formation of multilamellar vesicles (Figure 11) [62].

#### 2.6.7. The Enzymatic Method

In this strategy, niosomes are produced through an enzymatic route from a mixed micellar solution. Ester bond is sliced by esterases causing breakdown of products such as cholesterol and polyoxyethylene, which are in combination with dicetyl phosphate and other lipids that yield multilamellar niosomes. The surfactants used in this method are polyoxyethylene stearyl derivatives [63] and polyoxyethylene cholesteryl sebacetate diacetate [64].

#### 2.6.8. The Single-Pass Method

It is a patented method including an incessant procedure which contains the extrusion of a solution or suspension of lipids that concluded a porous device and subsequently through a nozzle. It associates homogenization and high-pressure extrusion to provide niosomes with a narrow size supply in the range 50–500 nm [65].

#### 2.6.9. Microfluidization

Microfluidization was a current strategy to give unilamellar vesicles of characterized estimate circulation. Based on the submerged jet principle, in this strategy, two fluidized streams connect at ultrahigh speeds, in correctly characterized smaller-scale channels inside the interface chamber. The impingement of thin-liquid sheet beside a common front was settled such that the energy delivered to the system remains within the area of niosomes establishment. The outcome was a more prominent consistency, reduced size, and well reproducibility of niosomes shape.

### 2.7. Separation of Unentrapped Drug

Several techniques were developed to achieve the removal of unentrapped solute from the vesicles such as dialysis, gel filtration, and centrifugation.

#### 2.7.1. Dialysis

Dialysis is the main technique used for removal of the unentrapped drug from vesicles. The aqueous niosomal dispersed was evaluated in dialysis tubing against phosphate buffer or normal saline or glucose solution [60].

#### 2.7.2. Gel Filtration

The unentrapped drug is uninvolved by gel filtration of niosomal dispersion through a Sephadex-G-50 column and elution with phosphate-buffered saline or normal saline [60].

#### 2.7.3. Centrifugation

The niosomal suspension was centrifuged, and the above phase was discarded. The pellet was resuspended to give a niosomal suspension free from unentrapped medication [66, 67].

### 2.8. Characterization of Niosomes

#### 2.8.1. Size and Vesicle Charge

Size and charge of vesicles played main role in their steadiness and drug encapsulation. Size and charge can be determined by a multifunctional zeta potential analysis, in which the size of vesicles was the result of repulsion forces between the bilayers and the entrapped drug. Size of vesicles can be resolute by electron microscopy, molecular sieve chromatography, ultracentrifugation, photon correlation, and optical and freeze fracture electron microscopy [54].

#### 2.8.2. Encapsulation Efficiency

Vesicles were digested with suitable organic solvents such as 50% *n*-propanol or 0.1% triton X-100 and examined with a suitable analytical method [63].

The encapsulation efficiency (EE) percentage is calculated according to the following equation:

(2) $$\begin{array}{c} \text{EE}\%=\frac{\text{amount of drug entrupment}}{\text{total amount of drug}}\times 100. \end{array}$$

#### 2.8.3. In Vitro Release Study

*In vitro* release studies are performed by release frequency that contains the use of dialysis tubing. The vesicle suspension was combined in an open-end dialysis membrane and placed in a receptor compartment comprising buffer solution with continuous shaking at 25°C or 37°C. Trials are sporadically collected and tested by approved procedures [51, 63, 68].

### 2.9. Stability

The major complications related to storing of vesicles are photodegradation, aggregation, fusion, and leakage of medication. Ammar et al. [69] reported a stable formulation of tenoxicam as these show high entrapment efficiency (>60%) and retention (>90%) above 30 days. After 30 days, only stable formulations were designated to remain for another 30 days. It was established that there is not significant modification in the size of vesicles after 90 days when equaled with those of newly set niosomes. However, the entrapment efficacy was reduced (10%) after storing [70].

### 2.10. Therapeutic Applications of Niosomes

Niosomes present an effective drug-delivery system with many pharmaceutical requests (Table 1). Some of them are labeled below.

#### 2.10.1. Protein and Peptide Delivery

Protein delivery after oral administration was restricted via several fences that include proteolytic enzymes, pH, and little epithelial permeability. Niosomes were applied to effectively keep the peptides from gastrointestinal collapse. Pardakhty et al. presented that the oral administration of rh-insulin as niosomal construction based on polyoxyethylene alkyl ethers was secure in contradiction with proteolytic action of chymotrypsin, trypsin, and pepsin. The drug release kinetics was defined by the Baker and Lonsdale equation indicating a diffusion-based delivery mechanism. Niosomes can be established as sustained release oral formulae for transport of peptides and proteins [38, 90].

#### 2.10.2. Transdermal Delivery

Although several drugs were explained for transdermal delivery, niosomes permeation into the skin is still problematic. The flexible noisome construction is an expectant approach to overcome the problem. Transdermal transport of NSAIDs can be the greatest way to escape gastric conflicts. Transfersomes and elastic niosomes are multipurpose kinds of vesicles for transdermal carriage [91]. Manosroi et al. [92] reported anti-inflammatory properties of gel comprising new flexible niosomes captured through diclofenac diethylammonium.

#### 2.10.3. Pulmonary Delivery

For asthmatic patients, inhalation treatment is the basis of cure; then, it is restricted by deprived infusion of medication over hydrophilic mucus. Terzano et al. [93] reported that beclomethasone dipropionate as niosomes-based polysorbate 20 was applied for prolonged obstructive pulmonary disease. They reported that the niosomes delivered sustained and targeted delivery, better mucus infusion, and improved therapeutic result.

#### 2.10.4. Carrier for Hemoglobin

Niosomes could be an important transporter for hemoglobin inside the blood. The niosomal vesicles are absorptive to oxygen, and therefore, it performs as a transporter for hemoglobin [94].

#### 2.10.5. Vaccine and Antigen Delivery

Some surfactants have immunostimulatory possessions and have been applied as vaccine adjuvants. The adjuvanticity of niosomes primed from 1-monopalmitoyl glycerol: cholesterol: dicetyl phosphate (5 : 4 : 1) was established in mice that administered a subcutaneous vaccination of ovalbumin or a synthetic peptide comprising a known T-cell epitope and bovine serum albumin [95, 96].

#### 2.10.6. Cancer Chemotherapy

In cancer chemotherapy, targeting with medication transporter system can be allocated into three forms, passive targeting, physical targeting, and active targeting (ligand mediated targeting and physical targeting).

*(1) Passive Targeting*. Passive targeting facilitates deposition of nanoparticles within the tumor microenvironment, due to particular features inherent to the tumor milieu, not normally existing in healthy tissues [96]. The delivery of nanoparticles was defined by numerous aspects such as tumor microvasculature, nanoparticle size, shape, and surface charge [96].

*(2) Physical Targeting*. It refers to delivery systems that release a drug only when exposed to a specific microenvironment such as a change in pH or temperature or the use of an external magnetic field.

*(3) Active Targeting*. It facilitates the active uptake of nanoparticles in the tumor cells themselves. It can engage the versatile molecules to functionalized medication vesicles to identify tumor tissue targets.

By modification of the carrier structure, several modifications are ensued such as change in the molecular size, adjustment of the surface properties, incorporation of antigen-specific antibodies, or attachment of cell receptor-specific ligands. Several ligand-targeting agents were used for brain drug delivery such as low-density lipoproteins, rabies virus glycoprotein (RVG29), transferrin receptor, insulin receptor, propionylated amylose helix, phosphatidylethanolamine, Apo E-reconstituted HDLs, ApoE3 porphyrin-lipid, Angiopep-2.

**Figure 12:**
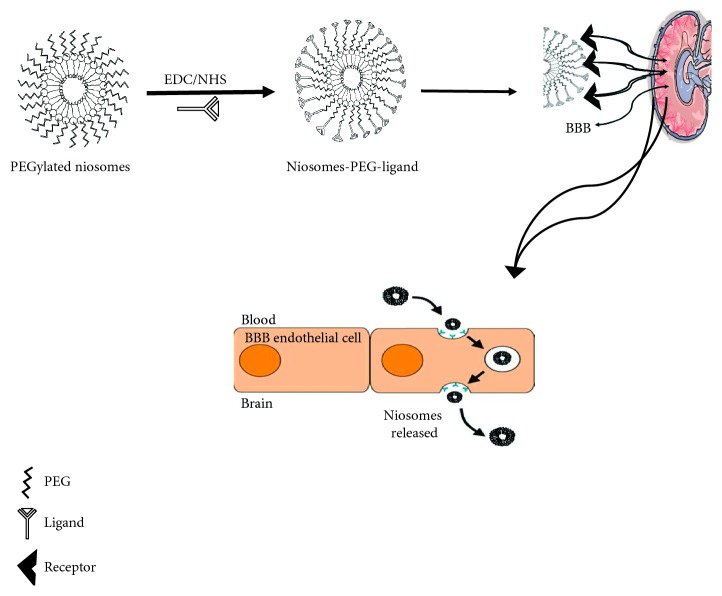
Schematic conjugation of targeting ligand to PEGylated niosomes delivery to BBB.

*(4) Active Targeting with Surface Engineered Niosomes, Functionalized with Targeting Ligands*. As it is well known, the structure of niosomes is similar to liposome in structure; thus, the surface-functionalized liposome methods can be used to functionalize surface niosomes. Two types of active targeting strategies are widely used for drug targeting to the desired organ/tissue. One of the strategies was that ligands for active targeting have been attached directly to the cholesterol or that ligand was devoted to the distal end of PEG chains in PEGylated niosomes. The other one, the traditional niosomes formulation method, was incorporation of the cholesterol-PEG-ligand conjugate, into the niosomes formulation step [97–99]. Preparation of PEGylated niosomes conjugated with each ligand is shown in Figure 12.

Several studies have been functionalized the niosomes with some of ligands such as glucose-targeted niosomes for transport of vasoactive intestinal peptide (VIP) [90], glucose derivative N-palmitoylglucosamine to develop as probable transporter for brain-targeted delivery of the neuropeptide DynB [100] and Doxorubicin [101]. Also, folic acid and transferrin-targeted niosomes have been made up as possible carrier for CNS drug delivery [102].

## 3. Conclusion

Nonionic surfactant vesicles were introduced as an innovative and capable method to natural drug delivery. They are mainly composed of nonionic surfactants and cholesterol, and their inside usually comprise a buffer solution at proper pH. They can be made by different approaches, which affect the establishment and the properties of the medication, cholesterol amount, structure, type, and amounts of surfactant. As a drug delivery method, niosomes are osmotically active, less toxic, and chemically stable. Surface modification is comparatively easy on them, due to the functional groups that can add on their hydrophilic heads. Niosomes active targeting to the desire tissue is arbitrated with several therapeutic means as ligand of the distinctive receptor. This system has an optimistic upcoming in pharmaceutical uses, mainly with the increasing availability of new schemes to overcome BBB and targeting the niosomes to the CNS.

## Figures and Tables

**Table 1:** Recent studies in drug delivery using niosomes

Application	Surfactant	Method	Drug	Route administration	Reference
Pulmonary delivery	Tween 60	Lipid layer hydration	Ciprofloxacin	Inhaler	[71]
	Span 60	Lipid layer hydration	Clarithromycin	Inhaler	[72]
	Span 60	Sonication	Rifampicin	Intratracheal	[73]

Protein delivery	Brij 92	Lipid layer hydration	Insulin	Oral	[74]
	Span 60	Lipid layer hydration	Insulin	Oral	[75]
	Span 40	Lipid layer hydration	N-acetyl glucosamine	Topical	[76]
	Span 60	Lipid layer hydration	Bovine serum albumin	Oral	[77]

Cancer chemotherapy	Span 60	Lipid layer hydration	Cisplatin		[78]
	Span 60	Lipid layer hydration	5-Flourouracil	Topical	[79]
	Span 80	Sonication	Curcumin		[79]
	Bola surfactant	Lipid layer hydration	5-Fluorouracil	Intravenous	[80]
	Span 60	Lipid layer hydration	5-Fluorouracil	Topical	[81]

Carrier for hemoglobin	Span 60	Lipid layer hydration	Hemoglobin	Intravenous	[82]

Treatment of HIV-AIDS	Span 60	Lipid layer hydration	Lamivudine		[83]
	Span 60	Ether injection	Stavudine		[84]
	Span 60	Lipid layer hydration	Stavudine		[31]
	Span 80	Eether injection	Zidovudine		[85]

Vaccine and antigen delivery	Span 60	Lipid layer hydration	Tetanus toxoid		[86]
	Span 20	Lipid layer hydration	Newcastle disease vaccine	Parenteral	[87]
	Span 60	Lipid layer hydration	Ovalbumin		[88]
	Span 60/span 85	Reversed- phase evaporation	Bovine serum albumin	Topical vaccine	[89]
